# Tuberculosis among economic migrants: a cross-sectional study of the risk of poor treatment outcomes and impact of a treatment adherence intervention among temporary residents in an urban district in Ho Chi Minh City, Viet Nam

**DOI:** 10.1186/s12879-020-4865-7

**Published:** 2020-02-12

**Authors:** Luan Nguyen Quang Vo, Andrew James Codlin, Rachel Jeanette Forse, Hoa Trung Nguyen, Thanh Nguyen Vu, Vinh Van Truong, Giang Chau Do, Lan Huu Nguyen, Giang Truong Le, Maxine Caws

**Affiliations:** 1 Friends for International TB Relief, 68B Nguyen Van Troi, 8, Phu Nhuan, Ho Chi Minh City, Viet Nam; 2 Interactive Research and Development, Ho Chi Minh City, Viet Nam; 3 Go Vap District Health Center, Ho Chi Minh City, Viet Nam; 4 Ho Chi Minh City Public Health Association, Ho Chi Minh City, Viet Nam; 5 Pham Ngoc Thach Hospital, Ho Chi Minh City, Viet Nam; 6 Liverpool School of Tropical Medicine, Department of Clinical Sciences, Liverpool, UK; 7 Birat Nepal Medical Trust, Lazimpat, Kathmandu, Nepal

**Keywords:** Adherence, Tuberculosis, Economic migrants, Treatment outcomes, Loss to follow-up, Impact evaluation

## Abstract

**Background:**

Tuberculosis (TB) remains a major cause of avoidable deaths. Economic migrants represent a vulnerable population due to their exposure to medical and social risk factors. These factors expose them to higher risks for TB incidence and poor treatment outcomes.

**Methods:**

This cross-sectional study evaluated WHO-defined TB treatment outcomes among economic migrants in an urban district of Ho Chi Minh City, Viet Nam. We measured the association of a patient’s government-defined residency status with treatment success and loss to follow-up categories at baseline and performed a comparative interrupted time series (ITS) analysis to assess the impact of community-based adherence support on treatment outcomes. Key measures of interest of the ITS were the differences in step change (β_6_) and post-intervention trend (β_7_).

**Results:**

Short-term, inter-province migrants experienced lower treatment success (aRR = 0.95 [95% CI: 0.92–0.99], *p* = 0.010) and higher loss to follow-up (aOR = 1.98 [95% CI: 1.44–2.72], *p* < 0.001) than permanent residents. Intra-province migrants were similarly more likely to be lost to follow-up (aOR = 1.86 [95% CI: 1.03–3.36], *p* = 0.041). There was evidence that patients > 55 years of age (aRR = 0.93 [95% CI: 0.89–0.96], *p* < 0.001), relapse patients (aRR = 0.89 [95% CI: 0.84–0.94], p < 0.001), and retreatment patients (aRR = 0.62 [95% CI: 0.52–0.75], *p* < 0.001) had lower treatment success rates. TB/HIV co-infection was also associated with lower treatment success (aRR = 0.77 [95% CI: 0.73–0.82], *p* < 0.001) and higher loss to follow-up (aOR = 2.18 [95% CI: 1.55–3.06], p < 0.001). The provision of treatment adherence support increased treatment success (IRR(β_6_) = 1.07 [95% CI: 1.00, 1.15], *p* = 0.041) and reduced loss to follow-up (IRR(β_6_) = 0.17 [95% CI: 0.04, 0.69], *p* = 0.013) in the intervention districts. Loss to follow-up continued to decline throughout the post-implementation period (IRR(β_7_) = 0.90 [95% CI: 0.83, 0.98], *p* = 0.019).

**Conclusions:**

Economic migrants, particularly those crossing provincial borders, have higher risk of poor treatment outcomes and should be prioritized for tailored adherence support. In light of accelerating urbanization in many regions of Asia, implementation trials are needed to inform evidence-based design of strategies for this vulnerable population.

## Background

Tuberculosis (TB) is a leading cause of death worldwide. In 2017, there were an estimated ten million incident cases of TB worldwide and 1.3 million TB deaths. An estimated 458,000 patients suffered from multidrug-resistant TB [1]. Viet Nam ranks 15th among the 30 high-burden countries, with 126,000 new TB patients each year, including 8200 rifampicin-resistant patients, and 13,000 deaths due to TB [2]. Through comprehensive implementation of the DOTS (Directly observed treatment, short-course) strategy, the Vietnamese National TB Control Program (NTP) has reduced TB prevalence and mortality by an average of 4–5% per annum [3].

In 2014, the government passed legislation to end TB by 2030 [4]. However, this ambitious goal will require sustained political commitment and multifactorial intensification of the TB program. For people with drug-susceptible TB, who comprise an estimated 95% of Viet Nam’s TB burden, the minimum treatment duration is 6 months and completing the full course of treatment can be challenging [5, 6]. Comprehensively documented barriers to successful treatment completion include pill burden, adverse events and the need for daily attendance at treatment clinics to take DOTS [7–9]. Another major reason is that adherence to TB treatment protocols represents a heavy economic burden for TB patients in Viet Nam and many other settings due to lost income, travel and opportunity costs [10, 11]. A key barrier to effective TB care and prevention is loss to follow-up (LTFU), which is a key contributor to drug resistance, continued transmission and death [12, 13]. Nevertheless, 77–92% of susceptible cases were successfully treated in 2017 [2].

Socially marginalized, high-risk subgroups commonly experience access barriers to TB care that result in poor treatment outcomes [14]. Economic migrants, defined as non-permanent residents in search of economic opportunity [15, 16], constitute a key affected population. These migrants may face systemic barriers to housing, education, financing and healthcare (Table 1) [17, 18]. As a result, they can suffer from a higher prevalence of TB compared to local residents and higher rates of treatment interruptions, poor outcomes and drug resistance [19, 20]. In addition to transiency, these populations also exhibit other characteristics that have been associated with poor treatment outcomes such as low socioeconomic status, high-risk behaviors and comorbidities [21, 22].

**Table 1:** Categorization of residents, their status, rights, obstacles and restrictions [17]

Category	Status	Rights	Obstacles/Legal restrictions
Permanent residents (KT1)	Residents (including both non-migrants and migrants) with permanent household registration at place of current residence	• Purchase and sell land and housing and have land/house ownership certificates • Access to public facilities and social services at current place of residence • Access to formal financial loans • Access to employment	• Access to public social services including education and health care only within their district of residence
Intra-province migrants (KT2)	Migrants who have permanent household registration in the province/city of current residence	• Purchase and sell land and housing and have land/house ownership certificates. • Access to public facilities and social services • Access to formal financial loans • Access to employment	• Access to education and health care only within the district where they are registered • Lack of access to financial loans/formal financial services
Long-term, inter-province migrants (KT3)	Migrants who do not have permanent registration at the place of current residence but have temporary registration for 6–12 months with the possibility of extension	• Access to public facilities and social services	• Lack of access to legal housing • KT3 children can go to public schools only when they are not used to full capacity (by KT1 and KT2 children). If the schools are overcrowded, KT3 children must attend private schools, where they have to pay higher school fees • Lack of access to financial loans/formal financial services
Short-term, inter-province migrants (KT4)	Migrants who do not have permanent registration at the place of current residence but have temporary registration for 1–6 months	Do not have the right to purchase land and access to public social services and financial loans	
Unregistered residents	Those who do not belong to any of the above categories	Do not have the right to purchase land and access to public social services and financial loans	

As for many Asian countries with a high TB burden, Viet Nam has undergone dramatic transformation in the last two decades. This resulted in rapid urbanization and economic migration, which may have also affected the local TB epidemiology. A study in Da Nang, an industrial hub in central Viet Nam, linked young, male migrants to increased notifications rates in urban, industrialized districts from 1999 to 2004 [23].

In the recent past, attention has been renewed in the potential of community health worker programs to accomplish public health objectives [24]. Studies across various disease areas have shown that treatment support provided by community health workers can improve treatment outcomes [25]. Despite heterogeneity in the engagement models, various studies have documented the positive influence of community-based groups on TB treatment outcomes [26, 27].

This study took place in Ho Chi Minh City (HCMC), one of the fastest growing cities of Viet Nam, and a magnet for rural-to-urban, economic migration. The city’s net migration rate of 11.6% over the past decade mirrored its average economic growth of over 11% per annum [28]. Low-income, inter-province migrants are estimated to settle in HCMC at a rate of approximately 200,000 people annually [29]. Unregistered migrants may raise population figures by 15% above official estimates [30]. In this context, we aimed to determine, if intensified treatment adherence support provided by community health workers can improve TB treatment outcomes in a city district with a high density of this vulnerable population.

## Methods

### Study design & aims

This is a cross-sectional analysis of routine TB surveillance data from Go Vap and District 8, Ho Chi Minh City, Viet Nam. The primary aim of the study was to determine if there is association between the government-defined residency status and WHO-defined treatment success and loss to follow-up. The secondary aim was to conduct a comparative impact evaluation on these two outcomes after initiation of a treatment adherence intervention carried out by community health workers in Go Vap. District 8 served as the concurrent control area providing only routine government TB program services.

### Study setting

**Fig. 1:**
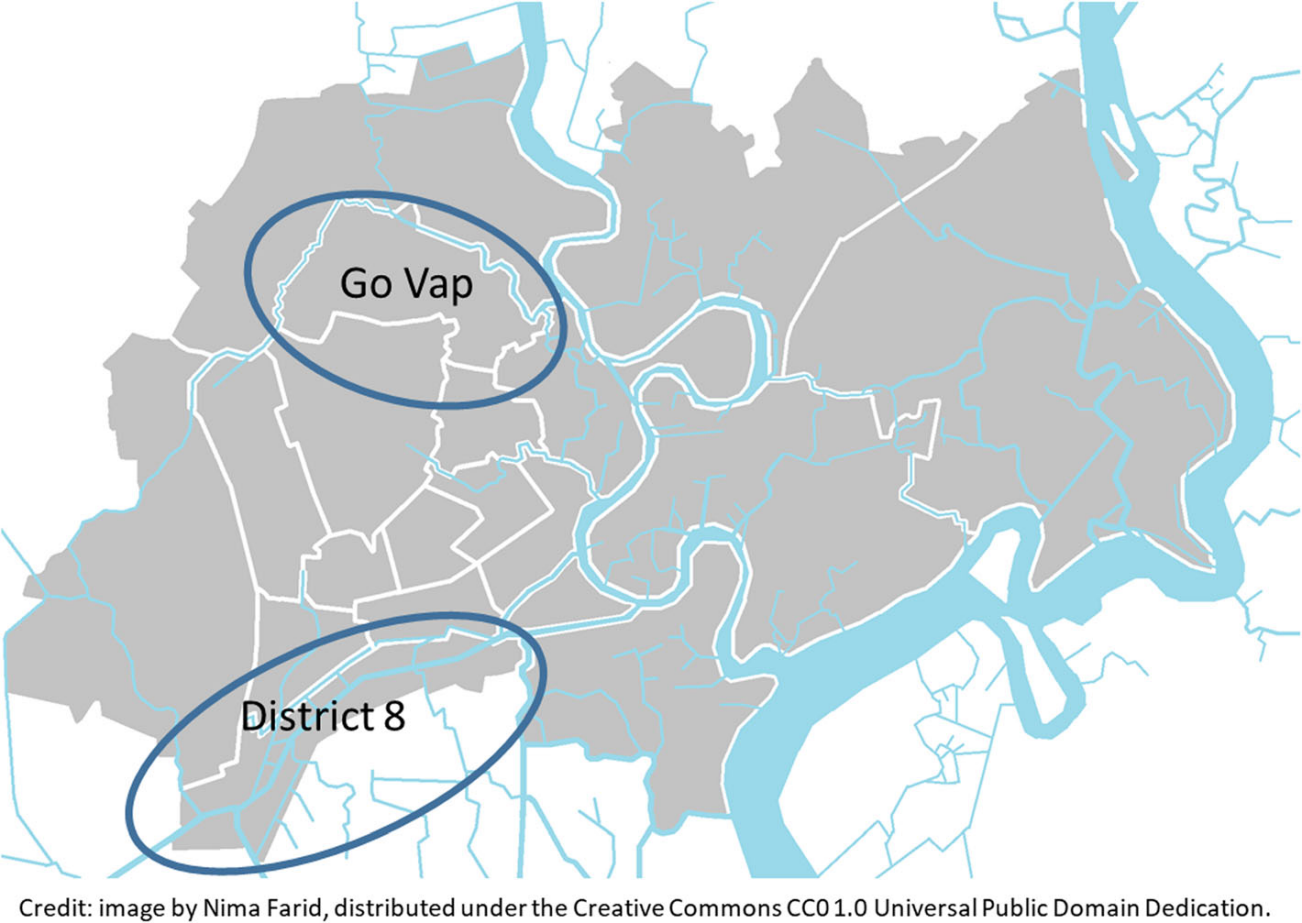
Relative location of Go Vap and District 8 in Ho Chi Minh City

Figure 1 shows the relative location of the two study districts within Ho Chi Minh City. Go Vap district housed a population of 685,000 people in 16 communes on an area of 20km^2^. The control was District 8, which housed 450,000 persons in 16 communes on 19km^2^ with a comparable demographic composition. Each district has one District TB Unit (DTU), which diagnoses TB and administers DOTS. The provincial TB control program recommended the intervention and control districts based on the comparability of their relative demographics and TB burden.

### Intervention

The intervention began in April 2014 and consisted of an intensified support program provided by incentivized community health workers. Individuals diagnosed with TB were contacted by the health workers within two weeks of diagnosis and received counseling at a location of their choice. Subsequent activities were determined by the support workers according to their perception of each patient’s need and included periodic in-person visits, phone calls or text messages with frequency and modality tailored to patient preferences and adherence patterns. Patients who missed scheduled appointments for directly observed therapy or follow-up sputum tests were contacted within 48 h by phone followed by a home visit by the community health worker, if unreachable.

### Community health workers

These activities were implemented by a cadre of 16 community health workers (CHW), who received a monthly salary (USD168) and performance-based incentives for case finding and treatment support. All CHWs were female with a median age of 56.5 (IQR: 54–58). The CHWs were recruited from local sociopolitical organizations, such as the Women’s Union and Red Cross Association, retired public health staff, and community health, population and family planning volunteers.

### Data sources & processing

The study used digitized data from patient registers of the NTP’s routine surveillance system. The sample consisted of all drug-sensitive TB patients notified by the Go Vap and District 8 DTUs from 1 January 2011 to 31 March 2017. The intervention commenced 31 March 2014. We excluded cases with missing data in any of the primary exposure or outcome variables. For the primary exposure we used Viet Nam’s official, four-tier residency classification system: 1) permanent resident (abbreviated KT1); 2) long-term-intra-province migrant (KT2); 3) long-term-inter-province migrant (KT3); and 4) short-term-inter-province migrant (KT4). Other available patient covariates from the registers were used to estimate secondary risk factors. To assess the post-intervention impact, we used monthly treatment success and loss to follow-up rates aggregated by treatment initiation date.

### Data analysis

All analyses were performed on Stata for Windows version 13. Descriptive statistics for study participants were cross-tabulated, and crude risk and odds ratios were calculated for primary and secondary exposures using univariate log-binomial and logistic regressions for treatment success and loss to follow-up, respectively.

Saturated, multivariate log-binomial and logistic maximum likelihood models were fitted onto the data to control for confounding and to identify pre-intervention risk factors. For non-converging log-binomial models we used Poisson regression with robust standard errors. We measured associations between treatment success and loss to follow-up rates, and individual parameters plus a binary bifurcation of permanent and temporary (KT2-KT4) residency for crude analyses. For multivariate analyses we used the categorical residency parameter, which we tested for a dose-response effect, considering short-term inter province migrants to be the most intensely exposed to the vulnerabilities of migrant status, and long-term intra-province migrants the least exposed.

We conducted a comparative interrupted time series (ITS) analysis on aggregate monthly treatment success and loss to follow-up rates using segmented log-linear Poisson regression with robust standard errors [31]. We modeled the ITS to include a step and a slope change. The step change aimed to reflect the instantaneous impact of the missed dose follow-up activities by the CHWs. The slope change estimated the impact of increased counseling and continuous case holding activities to cause gradual positive changes in treatment success and loss to follow-up rates. The parameters of the ITS were obtained for a segmented regression model with the following structure: *Y*_*t*_ = *β*_0_+*β*_1_*T*_*t*_ + *β*_2_*X*_*t*_ + *β*_3_*X*_*t*_*T*_*t*_ + *β*_4_*Z* + *β*_5_*ZT*_*t*_ + *β*_6_*ZX*_*t*_ + *β*_6_*ZX*_*t*_*T*_*t*_ + *ϵ*_*t*_. Here *Y*_*t*_ is the outcome measure along time t; *T*_*t*_ is the monthly time counter; *X*_*t*_ indicates pre- and post-intervention periods, *Z* denotes the intervention cohort, and *ZT*_*t*_, *ZX*_*t*_, and *ZX*_*t*_*T*_*t*_ are interaction terms. β_0_ to β_3_ relate to the control group as follows: β_0_, intercept; β_1_, pre-intervention trend; β_2_, post-intervention step change; β_3_, post-intervention trend. β_4_ to β_7_ represent differences between the control and intervention districts: β_4_, difference in baseline intercepts; β_5_, difference in pre-intervention trends; β_6_, difference in post-intervention step changes; β_7_, difference in post-intervention trend.

The ITS analysis was conducted in two iterations. The first iteration included the complete sample, while the second iteration focused on temporary residents. We included all patients notified after 1 August 2013 in the exposed group based on the proportion of treatment outcomes reported in the post-intervention period. The Cumby-Huizinga test was used to identify serial autocorrelation in the intervention district and to adjust the ITS analysis using the generalized estimating equation (GEE) approach. We obtained model specifications from quasi-likelihood information criteria. Hypothesis tests were two-sided and point estimates included 95% confidence intervals.

### Ethical considerations

The London School of Hygiene and Tropical Medicine Research Ethics Committee granted ethical approval for the epidemiologic analysis. The HCMC People’s Committee approved the implementation of the intervention. The Go Vap District Health Center approved use of the data. A consent waiver was granted based on the study’s use of routine surveillance data. We anonymized all patient data and removed identifying information prior to analysis.

## Results

The total sample included 10,515 drug-susceptible TB patients notified at the Go Vap and District 8 DTUs (Table 2), of whom 52.3% (5502/10,515) were notified prior to the intervention. The pre-intervention sample contained 31% (1711/5502) women and the median age was 41 (IQR: 29–53). Permanent residents comprised 77% (4258/5502) of the sample. Among temporary residents, 14% (170/1244) were classified as intra-province migrants, 27% (340/1244) held long-term, inter-province migrant status and 59% (734/1244) were registered as short-term, inter-province migrants. Treatment success was recorded for 84% of patients (4630/5502), while 5% (262/5502) were lost to follow-up. The overall death rate was 4% (232/5502), but ranged from 2% (25/1244) in temporary residents to 5% (207/4258) in permanent residents.

**Table 2:** Sample characteristics of notified TB cases by residency

	Total N (%)	Permanent residents, KT1 N (%)	Intra-province, KT2 N (%)	Long-term, inter-province KT3 N (%)	Short-term, inter-province KT4 N (%)	Temporary residents, KT2-KT4 N (%)
Total	5502 (100)	4258 (77)	170 (3)	340 (6)	734 (13)	1244 (23)
Sex						
Male	3791 (69)	3000 (70)	107 (63)	230 (68)	454 (62)	791 (64)
Female	1711 (31)	1258 (30)	63 (37)	110 (32)	280 (38)	453 (36)
Age						
< 25 years	829 (15)	529 (12)	29 (17)	62 (18)	209 (29)	300 (24)
25–34 years	1331 (24)	906 (21)	44 (26)	108 (32)	273 (37)	425 (34)
35–44 years	1123 (20)	904 (21)	33 (19)	65 (19)	121 (17)	219 (18)
45–54 years	1120 (20)	953 (22)	30 (18)	66 (19)	71 (10)	167 (13)
> 55 years	1087 (20)	955 (22)	34 (20)	39 (11)	59 (8)	132 (11)
Treatment outcome						
Success	4630 (84)	3587 (85)	137 (81)	295 (87)	611 (83)	1043 (83)
Cure	2598 (47)	2032 (48)	75 (44)	172 (51)	319 (43)	566 (46)
Complete	2032 (37)	1555 (37)	62 (36)	123 (36)	292 (40)	477 (38)
LTFU	262 (5)	179 (4)	13 (8)	9 (3)	61 (8)	83 (7)
Failure	225 (4)	188 (4)	8 (5)	16 (5)	13 (2)	37 (3)
Death	232 (4)	207 (5)	5 (3)	11 (3)	9 (1)	25 (2)
Transfer out	153 (3)	97 (2)	7 (4)	9 (3)	40 (5)	56 (5)
Patient type^┼^						
New	4301 (78)	3261 (21)	143 (84)	277 (81)	620 (84)	1040 (84)
Relapse	527 (10)	459 (11)	13 (8)	17 (5)	38 (5)	68 (5)
Failure	100 (2)	85 (2)	1 (1)	8 (2)	6 (1)	15 (1)
LTFU	55 (1)	44 (1)	2 (1)	2 (1)	7 (1)	11 (1)
Unknown	311 (6)	256 (6)	5 (3)	22 (6)	27 (4)	55 (4)
Transfer in	208 (4)	153 (4)	6 (4)	14 (4)	35 (5)	55 (4)
Type of TB^§^						
AFB(+)	3205 (34)	2500 (59)	94 (55)	208 (61)	403 (55)	705 (57)
AFB(−)	1096 (22)	862 (20)	38 (22)	66 (19)	130 (18)	234 (19)
EP	1201 (25)	896 (21)	38 (22)	66 (19)	201 (27)	305 (25)
HIV/AIDS^¶^						
No/Unknown	4886 (89)	3761 (88)	144 (85)	310 (91)	671 (91)	1125 (90)
Yes	616 (11)	497 (12)	26 (15)	30 (9)	63 (9)	119 (10)
Diabetes mellitus						
No/Unknown	5169 (94)	3976 (93)	160 (94)	321 (94)	712 (97)	1193 (96)
Yes	333 (6)	282 (7)	10 (6)	19 (6)	22 (3)	51 (4)

Notes

^┼^Failure = Retreatment after category I treatment failure; LTFU = Retreatment after loss to follow-up; Unknown = Retreatment with unknown/uncertain exposure to anti-TB drugs;

^§^AFB(+) = Sputum smear positive; AFB(−) = Sputum smear negative; EP = Extra-pulmonary TB;

^¶^Human Immunodeficiency Virus/Acquired Immunodeficiency Syndrome;

While crude analysis did not detect an association between treatment success and residency (Table 3), there was strong evidence that temporary residents (OR = 1.63 [95% CI: 1.25–2.13], *p* < 0.001) and particularly short-term, inter-province migrants (OR = 2.07 [95% CI: 1.53–2.79], *p* < 0.001) were more likely to be lost to follow-up in the pre-intervention period. Adjusting for potential confounders, short-term, inter-province migrants suffered marginally lower treatment success (aRR = 0.95 [95% CI: 0.92–0.99], *p* = 0.010), but were at substantially higher risk of loss to follow-up (aOR = 1.98 [95% CI: 1.44–2.72], *p* < 0.001) (Table 4). There was moderate evidence that intra-province migrants were more likely to be lost to follow-up (aOR = 1.86 [95% CI: 1.03–3.36], *p* = 0.041) than permanent residents.

**Table 3:** Crude associations of residency and secondary exposures with treatment success and loss to follow-up (*n* = 5502)

	Treatment success			Loss to follow-up		
	RR^‡^	95% CI	p-value^Þ^	OR^‡^	95% CI	p-value^Þ^
Total	n/a	n/a	n/a	n/a	n/a	n/a
Residency						
Permanent^¥^	1.00			1.00		
Temporary	1.00	[0.97, 1.02]	0.736	1.63	[1.25, 2.13]	< 0.001
Residency						
KT1^¥^	1.00			1.00		
KT2	0.96	[0.89, 1.03]	0.246	1.89	[1.05, 3.39]	0.033
KT3	1.03	[0.99, 1.08]	0.184	0.62	[0.31, 1.22]	0.167
KT4	0.99	[0.95, 1.02]	0.504	2.07	[1.53, 2.79]	< 0.001
Sex						
Male^¥^	1.00			1.00		
Female	1.05	[1.03, 1.08]	< 0.001	0.78	[0.59, 1.04]	0.089
Age						
< 25 years^¥^	1.00			1.00		
25–34 years	0.95	[0.91, 0.98]	0.001	1.23	[0.86, 1.78]	0.260
35–44 years	0.96	[0.92, 0.99]	0.015	0.84	[0.56, 1.26]	0.410
45–54 years	0.97	[0.94, 1.00]	0.071	0.58	[0.37, 0.91]	0.016
> 55 years	0.91	[0.87, 0.94]	< 0.001	0.55	[0.35, 0.86]	0.010
Patient type^┼^						
New^¥^	1.00			1.00		
Relapse	0.86	[0.82, 0.91]	< 0.001	1.02	[0.66, 1.58]	0.925
Failure	0.61	[0.51, 0.73]	< 0.001	1.61	[0.74, 3.52]	0.232
LTFU	0.82	[0.69, 0.97]	0.019	2.14	[0.84, 5.43]	0.109
Unknown	0.94	[0.89, 0.99]	0.018	1.39	[0.86, 2.26]	0.182
Transfer in	0.85	[2.05, 2.93]	< 0.001	1.66	[0.96, 2.87]	0.067
Type of TB^§^						
AFB(+)^¥^	1.00			1.00		
AFB(−)	1.09	[1.06, 1.12]	< 0.001	0.78	[0.55, 1.09]	0.143
EP	1.07	[1.04, 1.10]	< 0.001	0.87	[0.64, 1.20]	0.398
HIV/AIDS^¶^						
No/Unknown^¥^	1.00			1.00		
Yes	0.78	[0.74, 0.83]	< 0.001	2.56	[1.90, 3.46]	< 0.001
Diabetes						
No/Unknown^¥^	1.00			1.00		
Yes	0.96	[0.91, 1.01]	0.145	0.87	[0.50, 1.51]	0.622

Notes

^‡^Crude Risk Ratios and Odds Ratios calculated by univariate log binomial and logistic regression, respectively;

^Þ^Wald test;

^¥^Referent;

^┼^Failure = Retreatment after category I treatment failure; LTFU = Retreatment after loss to follow-up; Unknown = Retreatment with unknown/uncertain exposure to anti-TB drugs;

^§^AFB(+) = Sputum smear positive; AFB(−) = Sputum smear negative; EP = Extra-pulmonary TB;

^¶^Human Immunodeficiency Virus/Acquired Immunodeficiency Syndrome;

**Table 4:** Adjusted associations of residency and secondary exposures with treatment success and loss to follow-up (*n* = 5490)

	Treatment success		Loss to follow-up			
	aRR‡	95% CI	p-value^Þ^	aOR‡	95% CI	p-value^Þ^
Residency						
KT1^¥^	1.00			1.00		
KT2	0.95	[0.88, 1.02]	0.163	1.86	[1.03, 3.36]	0.041
KT3	1.01	[0.97, 1.05]	0.685	0.59	[0.30, 1.17]	0.134
KT4	0.95	[0.92, 0.99]	0.010	1.98	[1.44, 2.72]	< 0.001
Sex						
Male^¥^	1.00			1.00		
Female	1.02	[1.00, 1.05]	0.078	0.80	[0.60, 1.07]	0.139
Age						
< 25 years^¥^	1.00			1.00		
25–34 years	1.00	[0.97, 1.04]	0.881	1.00	[0.68, 1.46]	0.982
35–44 years	1.01	[0.97, 1.04]	0.622	0.73	[0.48, 1.11]	0.142
45–54 years	1.00	[0.96, 1.03]	0.863	0.59	[0.37, 0.93]	0.024
> 55 years	0.93	[0.89, 0.96]	< 0.001	0.58	[0.36, 0.93]	0.024
Patient type^┼^						
New^¥^	1.00			1.00		
Relapse	0.89	[0.84, 0.94]	< 0.001	1.09	[0.69, 1.73]	0.705
Failure	0.62	[0.52, 0.75]	< 0.001	1.77	[0.80, 3.95]	0.160
LTFU	0.87	[0.73, 1.02]	0.085	1.79	[0.69, 4.62]	0.228
Unknown	0.97	[0.92, 1.02]	0.198	1.42	[0.86, 2.36]	0.171
Transfer in	0.88	[0.81, 0.96]	0.003	1.48	[0.85, 2.60]	0.170
Type of TB^§,Π^						
AFB(+)^¥^	1.00			1.00		
AFB(−)	1.06	[1.03, 1.09]	< 0.001	0.79	[0.55, 1.12]	0.184
EP	1.05	[1.02, 1.08]	0.001	0.77	[0.55, 1.08]	0.126
HIV/AIDS^¶^						
No/Unknown^¥^	1.00			1.00		
Yes	0.77	[0.73, 0.82]	< 0.001	2.18	[1.55, 3.06]	< 0.001
Diabetes						
No/Unknown^¥^	1.00			1.00		
Yes	0.96	[0.91, 1.02]	0.159	1.15	[0.65, 2.03]	0.619

Notes

^‡^Adjusted summary RR and OR by log binomial and logistic regression, respectively, adjusted by all other variables listed in the table;

^Þ^Wald test;

^¥^Referent;

^┼^Failure = Retreatment after category I treatment failure; LTFU = Retreatment after loss to follow-up; Unknown = Retreatment with unknown/uncertain exposure to anti-TB drugs;

^§^AFB(+) = Sputum smear positive; AFB(−) = Sputum smear negative; EP = Extra-pulmonary TB;

^Π^not included in the final convergent multivariate model with LTFU as the primary outcome

^¶^Human Immunodeficiency Virus/Acquired Immunodeficiency Syndrome;

There was strong evidence that patients over 55 years of age (aRR = 0.93 [95% CI: 0.89–0.96], *p* < 0.001), relapse patients (aRR = 0.89 [95% CI: 0.84–0.94], *p* < 0.001), and retreatment patients (aRR = 0.62 [95% CI: 0.52–0.75], *p* < 0.001) were associated with lower treatment success. TB/HIV patients were associated with both lower treatment success (aRR = 0.77 [95% CI: 0.73–0.82], *p* < 0.001) and higher loss to follow-up (aOR = 2.18 [95% CI: 1.55–3.06], *p* < 0.001). Conversely, smear negativity (aRR = 1.06 [95% CI: 1.03–1.09], *p* < 0.001) and extra-pulmonary TB (aRR = 1.05 [95% CI: 1.02–1.08], *p* = 0.001) were associated with higher treatment success, while TB patients with 45–54 years of age (aOR = 0.59 [95% CI: 0.37, 0.93], *p* = 0.024) and 55+ years (aOR = 0.58 [95% CI: 0.36, 0.93], *p* = 0.024) were less likely to be lost to follow-up.

**Fig. 2:**
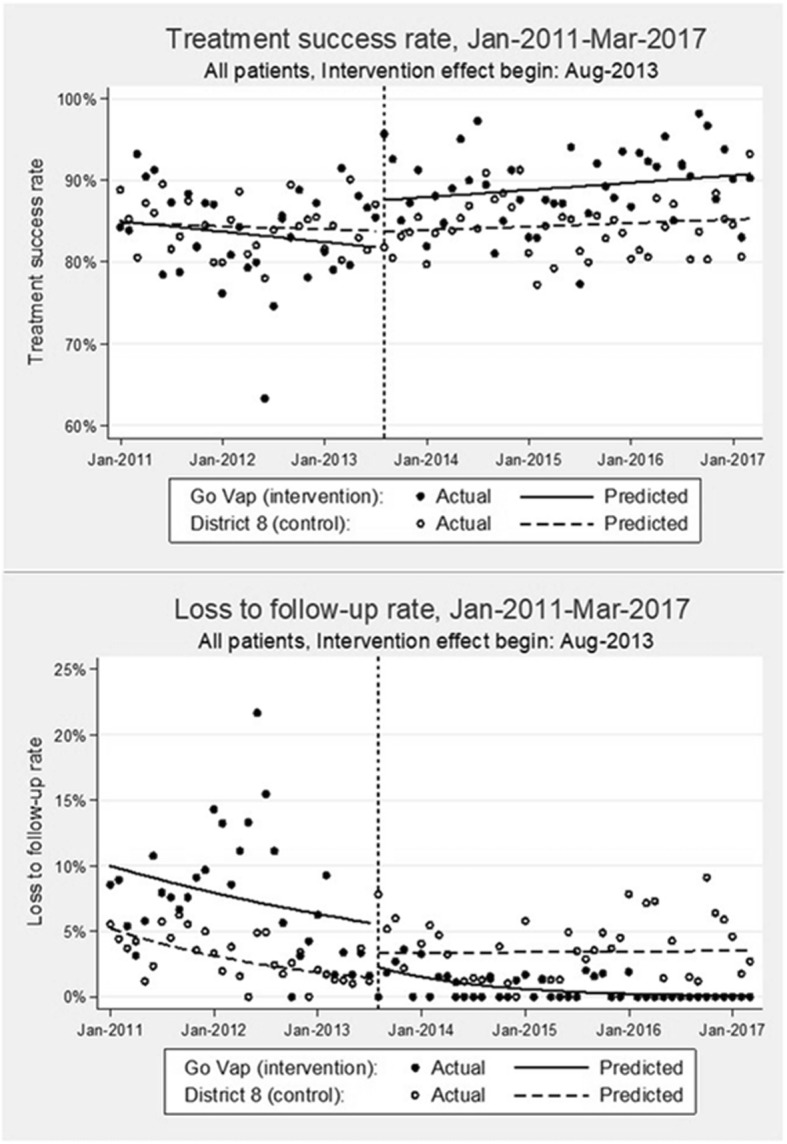
Comparative interrupted time-series analysis graphs for treatment success and loss to follow-up

The time series data consisted of 150 monthly aggregate counts of treatment outcomes balanced between intervention and control districts. The median number of monthly outcomes in both districts was 69 (IQR: 61–78) with a median treatment success count of 59 (IQR: 52–69) and a median loss to follow-up count of 2 (IQR: 0–4). There was no statistical difference between the intervention and control districts (Table 5 and Fig. 2) in baseline rate (β_4_) and pre-intervention trend (β_5_) for either outcomes of interest, i.e., treatment success (p(β_4_) = 0.909; p(β_5_) = 0.541) and loss to follow-up (p(β_4_) = 0.060; p(β_5_) = 0.305). After implementation of the intervention and adjusting for trends in the control area, we measured a step increase in treatment success (IRR(β_6_) = 1.07 [95% CI: 1.00, 1.15], *p* = 0.041) and a step reduction in loss to follow-up (IRR(β_6_) = 0.17 [95% CI: 0.04, 0.69], *p* = 0.013). We further detected evidence of a significant trend change in the control-adjusted, post-intervention loss to follow-up rate (IRR(β_7_) = 0.90 [95% CI: 0.83, 0.98], *p* = 0.019) in the intervention district. We did not detect a statistical difference in post-intervention treatment outcomes among temporary residents.

**Table 5:** Comparative interrupted time series analysis of monthly treatment success and loss to follow-up rates

	Treatment success			Loss to follow-up		
	IRR┼	95% CI	p-value^Þ^	IRR‡	95% CI	p-value^Þ^
Baseline rate (*β*_*0*_)¥	0.85	[0.83, 0.87]	< 0.001	0.05	[0.03, 0.09]	< 0.001
Pre-intervention trend, control (*β*_*1*_)	1.00	[1.00, 1.00]	0.624	0.96	[0.92, 0.99]	0.024
Post-intervention step change, control (*β*_*2*_)	1.00	[0.97, 1.03]	0.971	2.41	[0.97, 6.00]	0.059
Post-intervention trend, control (*β*_*3*_)	1.00	[1.00, 1.00]	0.382	1.04	[1.00, 1.09]	0.050
Difference in baseline (*β*_*4*_)	1.00	[0.95, 1.05]	0.909	1.91	[0.97, 3.76]	0.060
Difference in pre-intervention trends (*β*_*5*_)	1.00	[1.00, 1.00]	0.541	1.02	[0.98, 1.07]	0.305
Difference in post-intervention step change (*β*_*6*_)	1.07	[1.00, 1.15]	0.041	0.17	[0.04, 0.69]	0.013
Difference in post-intervention trends (*β*_*7*_)	1.00	[1.00, 1.00]	0.435	0.90	[0.83, 0.98]	0.019

Notes

All patients in intervention and control districts, January 2011 to March 2017

^¥^The parameters were obtained for a segmented regression model with the following structure: *Y*_*t*_ = *β*_0_ + *β*_1_*T*_*t*_ + *β*_2_*X*_*t*_ + *β*_3_*X*_*t*_*T*_*t*_ + *β*_4_*Z* + *β*_5_*ZT*_*t*_ + *β*_6_*ZX*_*t*_ + *β*_6_*ZX*_*t*_*T*_*t*_;+*ϵ*_*t*_. Here *Y*_*t*_ is the outcome measure along time t; *T*_*t*_ is the monthly time counter; *X*_*t*_ indicates pre- and post-intervention periods, *Z* denotes the intervention cohort, and *ZT*_*t*_, *ZX*_*t*_, and *ZX*_*t*_*T*_*t*_ are interaction terms. β_0_ to β_3_ relate to the control group as follows: β_0_, intercept; β_1_, pre-intervention trend; β_2_, post-intervention step change; β_3_, post-intervention trend. β_4_ to β_7_ represent differences between the control and intervention districts: β_4_, difference in baseline intercepts; β_5_, difference in pre-intervention trends; β_6_, difference in post-intervention step changes; β_7_, difference in post-intervention trend

^┼^IRR based on log-linear Poisson regression with robust standard error estimations;

^‡^IRR based on log-linear GEE Poisson regression with an autoregressive correlation structure with lag order 2;

^Þ^Wald test;

## Discussion

Our findings suggest that under routine program conditions, economic migrants in Go Vap and District 8, particularly those crossing provincial borders, suffered poorer TB treatment outcomes. These findings are concordant with past studies in other countries that evinced greater rates of non-adherence, challenges in case management and higher rates of LTFU among economic migrants [32, 33]. While most prior research dichotomized study populations into local residents and rural-to-urban migrants [34], we aimed to increase granularity by identifying the relative risks within Viet Nam’s four official residency designations including three sub-segments of temporary residents. While there was no clear dose-response relationship, two of the three temporary resident subgroups indeed experienced poorer treatment outcomes. Particularly, short-term-inter-province (KT4) migrants – the most vulnerable subgroup of temporary residents in terms of access to public services (Table 1) – exhibited the highest risk and strongest statistical evidence for lower treatment success and higher loss to follow-up.

Several secondary factors were similarly strong predictors of poor treatment outcomes. HIV co-infection, prior history of TB and old age were associated with lower treatment success. These findings are concordant with other settings [35, 36]. This result also explains the higher death rate among permanent residents. Economic migrants tend to be younger as their main objective for migration is livelihood improvement [15]. This usually excludes older and sick populations from this group and therefore renders the group less likely to die throughout the course of TB treatment. In our study, smear negative and extra-pulmonary TB patients showed a higher likelihood of treatment success. The higher treatment success in these patients was possibly a result of the Go Vap DTU’s decentralization strategy for DOT. While smear positive TB patients took TB treatment under DOT at the DTU until the end of the intensive phase, smear negative and extra-pulmonary patients were referred to their commune health station immediately upon enrollment. The shorter distances for DOT may have contributed to higher adherence and better outcomes as observed in other settings [37, 38].

In addition to poorer treatment outcomes, economic migrants have exhibited an elevated risk profile along the entire TB care pathway. Economic migrants tend to be of lower socioeconomic status, suffer from poor living conditions and experience challenges in access to healthcare in general [39] and TB care services in particular [40]. Access barriers contribute to patient- and provider-initiated delays in health-seeking and in diagnosis of the disease [41], whereby TB infection may also be more prevalent among economic migrants [42]. With rising urbanization, these economic migrants constitute an escalating risk factor to successful TB care and prevention. In addition, studies that assessed the impact on the point of origin of economic migrants found elevated risk of “exported” TB among family members of these circular migrants. This mobility and geographic reach further exacerbates the complexity of TB care in this subpopulation [43].

Our comparative ITS analysis was concordant with previously reported findings that community-based treatment support can have a substantial, positive effect on treatment outcomes [44]. As prior evidence has shown, the use of incentives and subsidies, and support for side effect management can also have a significant positive impact on TB treatment outcomes in urban migrants [16, 45]. Even though our comparative ITS analysis on the subpopulation of temporary residents failed to detect a significant change after implementation of the intervention possibly due to data sparsity, it may be reasonable to extend our findings to economic migrants given their high proportion in the general population (~ 38%) and TB patient cohort (23%) of Go Vap. The disparity in these two proportions also reinforces the WHO recommendation of systematic screening among migrants, as studies have shown that intensified case finding along with patient education, advocacy and robust referral mechanisms can have a positive impact on detection within this vulnerable group [46].

Research on TB and migration has traditionally concentrated on international cross-border migration, particularly from high-incidence, low-resource settings to high-income countries [47]. Economically-motivated, rural-to-urban migration has become an area of interest only in recent years with rising urbanization trends. The relative scarcity of studies on these rural-to-urban migrants is possibly due to insufficiently recorded residency status outside of countries with strong central planning and unitary political systems, such as China and Viet Nam, where institutionalized household registration systems (*hokuo* and *ho khau*, respectively) can facilitate identification and segmentation of the general population into these subgroups. However, even in the absence of clear denomination mechanisms, studies from developed countries have shown the effectiveness of social support in improving TB treatment outcomes among migrants [48]. Nevertheless, more evidence is needed to address potential barriers to economic migrants in registering residency, gaining full access to locally available public services and overcoming the risk inequalities in this subpopulation tied to social determinants of health.

The study presented here suffers from several limitations. The data may contain measurement bias due to its reliance on routine surveillance data and due to low sample sizes in certain subgroups such as the monthly number of temporary residents notified and subsequently lost to follow-up. The cross-sectional nature of the study limits the ability to infer causality and generalizability of the results. Nevertheless, this study may serve as an initial example of further stratification of internal migrants for more detailed analyses, and design of bespoke interventions and policy responses.

## Conclusions

Ending TB will require comprehensive understanding and intervention among key affected populations. Economic migrants represent one such vulnerable population that suffer from greater risk of TB and higher likelihood of poor treatment outcomes. While this study showed that community-based support can be an appropriate response for this vulnerable group, more tailored research specific to economic migrants is needed to understand the root causes and develop appropriate policy responses and protection mechanisms.

## Abbreviations

(a)OR: (adjusted) Odds Ratio
(a)RR: (adjusted) Rate Ratio
AFB: Acid-fast Bacilli
AIDS: Acquired Immunodeficiency Syndrome
CHW: Community Health Worker
CI: Confidence Interval
DOT(S): Directly observed treatment (short-course)
DTU: District TB Unit
EP: Extra-pulmonary
GEE: Generalized Estimating Equation
HCMC: Ho Chi Minh City
HIV: Human Immunodeficiency Virus
IQR: Interquartile Range
IRR: Incidence Rate Ratio
ITS: Interrupted Time Series
KT1: Permanent resident
KT2: Long-term-intra-province migrant
KT3: Long-term-inter-province migrant
KT4: Short-term-inter-province migrant
LTFU: Loss to follow-up
NTP: National TB Control Program
TB: Tuberculosis
WHO: World Health Organization
